# Indications for Three Independent Domestication Events for the Tea Plant (*Camellia sinensis* (L.) O. Kuntze) and New Insights into the Origin of Tea Germplasm in China and India Revealed by Nuclear Microsatellites

**DOI:** 10.1371/journal.pone.0155369

**Published:** 2016-05-24

**Authors:** M. K. Meegahakumbura, M. C. Wambulwa, K. K. Thapa, M. M. Li, M. Möller, J. C. Xu, J. B. Yang, B. Y. Liu, S. Ranjitkar, J. Liu, D. Z. Li, L. M. Gao

**Affiliations:** 1 Key Laboratory for Plant Diversity and Biogeography of East Asia, Kunming Institute of Botany, Chinese Academy of Science, Kunming 650201, China; 2 Germplasm Bank of Wild Species in Southwest China, Kunming Institute of Botany, Chinese Academy of Science, Kunming 650201, China; 3 University of Chinese Academy of Science, Beijing 10049, China; 4 Coconut Research Institute, Lunuwila, Sri Lanka; 5 World Agroforestry Centre, Nairobi, Kenya; 6 Department of Botany, Dinhata College, Dinhata– 736135, West Bengal, India; 7 Royal Botanic Garden Edinburgh, 20A Inverleith Row, Edinburgh EH3 5LR, Scotland, United Kingdom; 8 Centre for Mountain Ecosystem Studies and World Agroforestry Centre East and Central Asia Regional Office, Kunming Institute of Botany, Chinese Academy of Science, Kunming 650201, China; 9 Tea Research Institute of Yunnan Academy of Agricultural Sciences, Menghai 666201, China; Washington University, UNITED STATES

## Abstract

**Background:**

Tea is the world’s most popular non-alcoholic beverage. China and India are known to be the largest tea producing countries and recognized as the centers for the domestication of the tea plant (*Camellia sinensis* (L.) O. Kuntze). However, molecular studies on the origin, domestication and relationships of the main teas, China type, Assam type and Cambod type are lacking.

**Methodology/Principal Findings:**

Twenty-three nuclear microsatellite markers were used to investigate the genetic diversity, relatedness, and domestication history of cultivated tea in both China and India. Based on a total of 392 samples, high levels of genetic diversity were observed for all tea types in both countries. The cultivars clustered into three distinct genetic groups (i.e. China tea, Chinese Assam tea and Indian Assam tea) based on STRUCTURE, PCoA and UPGMA analyses with significant pairwise genetic differentiation, corresponding well with their geographical distribution. A high proportion (30%) of the studied tea samples were shown to possess genetic admixtures of different tea types suggesting a hybrid origin for these samples, including the Cambod type.

**Conclusions:**

We demonstrate that Chinese Assam tea is a distinct genetic lineage from Indian Assam tea, and that China tea sampled from India was likely introduced from China directly. Our results further indicate that China type tea, Chinese Assam type tea and Indian Assam type tea are likely the result of three independent domestication events from three separate regions across China and India. Our findings have important implications for the conservation of genetic stocks, as well as future breeding programs.

## Introduction

Plant domestication has shaped human history over the past 13,000 years, accelerating the codependence between human societies on the one hand and plants on the other [1, 2]. It is suggested that the domestication of food crops evolved independently in 24 regions among hunter-gatherer communities worldwide, of these 13 early cultivation centers were solely for grain crops, among which three were located in China including northern China, Central China (Yangtze river region) and the Himalayas & Yunnan uplands, with India representing another [3]. Initially, domesticated crops comprised mostly annuals while long-lived perennial and tree crops were domesticated only later [4]. For annuals, specific traits selected for during domestication included large fruit / grain size [5], reduced seed shattering [6], compact inflorescence structure [7], and compact plant architecture [8]. While for long-lived perennials and tree crops, such as tea and coffee, secondary metabolites were the primary sources of variation that were selected for during their domestication [9]. Some food crops, such as common bean and coconut, were domesticated multiple times [10,11], while many others, such as rice, maize, sunflower, potato, olive, and grapes this occurred only once [12–17].

The tea plant, *Camellia sinensis* (L.) O. Kuntze, is an ancient tree crop highly regarded as the oldest and most popular nonalcoholic beverage [18, 19]. It is grown in over 52 countries in tropical and subtropical regions around the world and is an important cash crop in many developing countries. China and India are ranked first and second tea producing countries in the world and account for 36.28% and 22.61% of the global tea production, respectively [20]. The taxonomy of the commercial tea plant is often debated [21–23]. Based on the classification of Wight [22], cultivated tea is recognized as 1) *Camellia sinensis* (L.) O. Kuntze possessing smaller leaves and is commonly known as China type; 2) *C*. *assamica* (Masters) Chang with larger leaves, commonly referred to as Assam type which was later treated as a variety of *C*. *sinensis* (i.e. *C*. *sinensis* var. *assamica*) [23,24]; and 3) *C*. *assamica* subsp. *lasiocalyx* Planch with medium-sized leaves and usually known as Cambod type [22,25], later treated as synonym of *C*. *sinensis* var. *assamica* [23]. China type trees are cultivated across South China and in some Southeast Asian countries. Assam type is cultivated similarly widely but is also grown in India and other tea growing countries across the globe, although in China it is restricted to the province of Yunnan. Cambod type tea was originally cultivated only in Indo-China (South Yunnan of China, Myanmar, Assam in India, Nothern Thailand, Vietnam, Laos and Cambodia), but today is produced worldwide.

It is undisputed that China tea originated in China and is thought to have been used for the first time as a medicine and later as a beverage as early as 2737 BC [26]. However, since no wild populations of the tea plant have ever been found, the exact species used for the first domestication of tea plants remains unknown [27]. The area of origin and domestication of tea in China have long been controversial. For example, the Sichuan province of China has been proposed as an area of origin according to the first monograph on the tea plant, "The classic of tea" by Lu [28]. While Stuart inferred that the mountain range between Yunnan in Southwest China and Assam in India was a possible location [29]. The area where Southwest China, Indo-Burma, and Tibet meet was also suggested as a possible place of origin of tea [30]. In addition, a large-leaved tea morphologically similar to Assam type tea (firstly described as *C*. *assamica* from Assam, India, in 1823 [31]) has been cultivated and used in the Yunnan province for over a thousand years [32]. Takeo et al. [33] speculated that Assam type tea was dispersed from Yunnan to central China along the Yangtze River over a thousand years ago, from which then China type tea was developed. Although many authors have postulated on the origin and early domestication of tea cultivars, no study has focused on these questions using molecular data.

To aid future breeding programs and conserve genotypes of tea cultivars, germplasm collections have been assembled including local cultivars and more recently germplasm has been exchanged between the main tea producing countries. A number of studies have assessed the genetic diversity and population structure of tea germplasm collections in China [32, 34–41] and India [42–47], but only a few studies have analyzed the genetic relationships of cultivated teas across tea producing countries [48,49] and most have confined the analyses to tea germplasm originating from the respective countries. Very few studies have investigated the domestication history of cultivated tea and the center of tea domestication, and the subject has remained unclear and controversial to date. In the present study, we use nuclear microsatellite markers to investigate the genetic diversity, relatedness, and domestication history of 392 tea plants collected in China and India. We focus particularly on the area of origin of tea plants and whether domestication is the result of single or multiple events and whether the big-leaved Assam type tea in China and India are the same or the result of independent domestication events.

## Materials and Methods

### Ethical Statement

The *Camellia sinensis* cultivars used in the current study are not an endemic or endangered species and authorization for their collection had been granted by the diverse germplasm centers.

### Plant materials

Out of the 392 samples that were collected from China and India for this study, 300 samples came from China, covering 14 main tea growing provinces (Yunnan, Guizhou, Sichuan, Guangdong, Guangxi, Fujian, Zhejiang, Anhui, Jiangxi, Hunan, Hubei, Henan, Jiangsu, Shandong). The samples from China consisted of 137 *C*. *sinensis* var. *sinensis* (China type tea) and 163 *C*. *sinensis* var. *assamica* (Assam type tea), and included 76 ancient trees of China type tea and Assam type tea from Yunnan and Guizhou (Table 1, S1 Table and S1 Fig). The 92 Indian tea samples included 41 China type tea, 45 Assam type tea, and 6 samples of *C*. *assamica* subsp. *lasiocalyx* (Cambod type tea). In the current study, the samples collected from China and India were denoted as follows: China type tea from China—CTC; Assam type tea from China—ASTC; China type tea from India—CTIN; Assam type tea from India—ASTIN and Cambod type tea as CAM. Leaf material of each sample was collected and dried in silica gel immediately upon collection. Vouchers were deposited in the herbaria of Kunming Institute of Botany (KUN), Chinese Academy of Sciences.

**Table 1:** Genetic diversity parameters of five tea types from China and India based on original assignments used in this study.

Country	Variety (Abbreviation of types)	N	*A*	*Ar*	*Ap*	*Ho*	*He*	*F*is
China	*C*. *sinensis* var. *sinensis* (CTC)	137	255	4.56	19	0.642	0.700	0.086
	*C*. *sinensis* var. *assamica* (ASTC)	163	269	4.46	36	0.579	0.701	0.176
India	*C*. *sinensis* var. *sinensis* (CTIN)	41	201	4.85	6	0.695	0.760	0.098
	*C*. *sinensis* var. *assamica* (ASTIN)	45	194	4.5	4	0.686	0.726	0.067
	*C*. *assamica* subsp. *lasiocalyx* (CAM)	6	108	4.36	0	0.729	0.644	-0.039
Total		392	315	5.125		0.666	0.706	0.078

N: number of samples; *A*: number of alleles; *Ar*: Allelic richness; *Ap*: number of private alleles; *Ho*: observed heterozygosity; *He*: expected heterozygosity; *F*is, inbreeding coefficient.

### DNA extraction and SSR genotyping

Total genomic DNA was extracted from each sample following a modified CTAB method [50]. We used the 23 primers showing polymorphisms in our previous studies [51,52] for all 392 samples genotyped in this study (S2 Table). PCR was carried out with a TaKaRa Taq^TM^ kit (TAKARA BIO INC., Dalian, China) in 20μl reactions that contained 1μl template DNA (50ng of total DNA), 2μl of PCR buffer, 1.6μl of 25mM MgCl_2_, 0.4μl of 10mM dNTPs, 0.4μl each of 5mM forward and reverse primers, 0.75 units of Taq polymerase and 14.05μl ddH_2_O. Each forward primer was 5'-end labeled with either FAM, TAM or HEX fluorescent dyes (Applied Biosystems, Foster City, CA, USA). PCR was carried out using a Veriti 96 well Thermocycler (Life Technologies, Carlsbad, USA) using the following profile: 94°C for 3 min, 35 cycles of 94°C for 30s, 53–65°C for 45s (depending on primer pair, S2 Table), 72°C for 1 min and a final extension of 72°C for 10 min. The PCR products were analyzed on an ABI 3730xl DNA sequencer.

### Data Analysis

The output profiles from the sequencer were manually checked and the fragment sizes recorded using GeneMarker v.2.2.0 (Applied Biosystems). MICROCHECKER [53] was used to find possible genotyping errors such as stuttering, large allele drop out and null alleles. Using the genotype data for all 23 SSR loci, the observed heterozygosity (*H*o), expected heterozygosity (*H*e) and private alleles (*Ap*) for each tea type were calculated in GenA1Ex v.6.5b4 [54]. Total allele number (*A*) for each population, allele richness (*A*r) and inbreeding coefficient (*F*is) were estimated using FSTAT v.2.9.3.2 [55].

To investigate the genetic structure among the samples, we used STRUCTURE [56] without prior grouping assumptions. In this analysis, we used the admixture model and evaluated 1 to 10 genetic clusters (*K*) with 20 permutations for each *K* value. STRUCTURE was run with 100,000 generations of burn-in followed by 100,000 Markov Chain Monte Carlo (MCMC) iterations. The optimal number of genetic clusters (*K*) was obtained using the method of Evanno [57] as implemented in STRUCTURE HARVESTER web v.0.6.94 [58]. Furthermore, the *K* value was determined by a Log-likelihood method obtained with STRUCTURE HARVESTER.

To ensure that the samples were assigned to the accurate tea types, all samples were placed in groups based on the results of the STRUCTURE analysis. We followed an admixture coefficient of ≥ 80% for assigning individuals to "pure" groups [16,47,52,59,60], while those with an intermediate admixture coefficient < 80% were consigned to a ‘Mosaic’ group. The newly formed groups were denoted as the "China tea", "Chinese Assam tea", "India Assam tea" and “Mosaic”. After assigning individuals into groups, we recalculated the observed heterozygosity (*Ho*), expected heterozygosity (*He*), private alleles (*Ap*), total allele number (*A*), allele richness (*Ar*), and inbreeding coefficient (*F*is) as explained above. Pairwise genetic differentiation (*F*st) among the tea groups was also calculated using FSTAT. A Principal Coordinate Analysis (PCoA) was carried out with GenA1Ex based on Nei’s genetic distances [61] to present genetic relationships among the tea groups. We constructed a UPGMA tree using Nei’s genetic distances for the regrouped dataset samples from China and India, excluding the individuals of the Mosaic group using MSA v.4.05 [62] with 1,000 multiple runs followed by 1,000 bootstrap replications in Phylip v.3.67 [63]. The UPGMA tree was viewed with FigTree v.1.4 [64].

## Results

### Genetic diversity

Out of the 23 primers that were used to screen the 392 tea samples from China and India, for ten primers (Po9, Ca8, A47, A87, TUGMS2-135, TUGMS2-143, TM179, TM197, S34, and S80) complete datasets were obtained, while the remaining 13 primers had 0.3 to 3.3% missing data due to failed PCR amplifications. No evidence of scoring errors due to large allele dropout or stuttering at any locus was detected. A total of 315 alleles were found, and the number of alleles per locus ranged from 8 (TUGMS2-157) to 22 (Q6) with an average of 13.7 per locus.

The highest expected heterozygosity (*He* = 0.760) and the second highest observed heterozygosity (*Ho* = 0.695) was shown for CTIN samples, followed by those of ASTIN (0.726 and 0.686) (Table 1). Interestingly, the CTC and ASTC samples exhibited a similar expected heterozygosity (0.701 and 0.700). The lowest expected heterozygosity (0.644) was estimated for CAM samples, while they exhibited the highest observed heterozygosity (0.729). Similarly, the highest allelic richness (*Ar*) was recorded for the CTIN accessions (4.85), followed by those of CTC (4.56), while the CAM samples showed the lowest value (4.36). The ASTC accessions had the highest number of alleles (*A* = 269) and private alleles (*Ap* = 36), followed by those of CTC with 255 (*A*) and 19 (*Ap*), respectively. The lowest number of alleles was found for the CAM samples (108) where no private alleles were detected (Table 1).

### STRUCTURE analysis and genetic grouping

**Fig 1:**
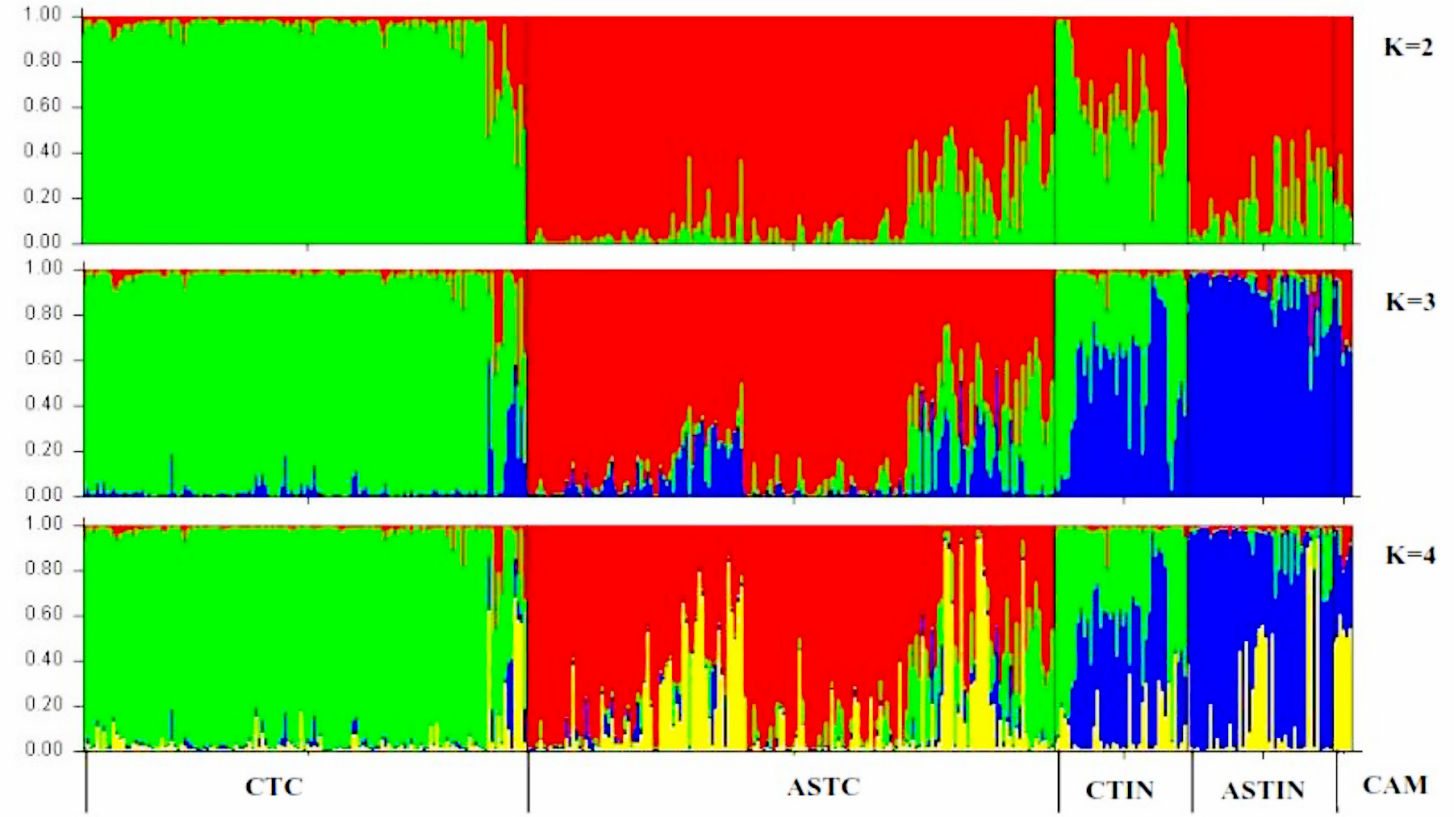
Results of the STRUCTURE analysis at *K* = 2 to 4 for a total of 392 tea samples collected from China and India assigned according to their respective types based on morphological characterization.

The best *K* value was suggested to be 2 and 3 based on *ΔK* and Log-likelihood K respectively (S2A Fig and S2B Fig). The results of the STRUCTURE analysis with the "admixture model" are given in Fig 1, showing *K* as 2, 3 and 4 respectively. With *K* = 2, all tea samples from China and India were assigned into two groups. One represented China type tea from both China and India and the other contained the Assam type tea from both countries plus the Cambod type tea from India. Most accessions of CTIN and some accessions of CTC had genetic components of both groups, and were similar to some cultivated tea plant accessions of ASTC, all CAM and most of ASTIN. With *K* = 3, accessions of CTC and ASTC formed two distinct genetic groups consistent with results of *K* = 2, while the accessions of ASTIN and CAM formed a third group. Most accessions of CTIN, especially the samples from Darjeeling of India, showed a mixed genetic composition of CTC and ASTIN + CAM. With *K* = 4, no additional distinct genetic groups were formed, although the group heterogeneity increased (Fig 1). Collectively, these results indicate that *K* = 3 is likely the best clustering solution for the current dataset.

When the 392 samples were regrouped based on an admixture coefficient of ≥ 80% (Qi ≥ 0.8) at *K* = 3, three distinct groups were found, namely the China tea group, the Chinese Assam tea group and the Indian Assam tea group, and additionally a Mosaic group. The STRUCTURE results after regrouping are given in S3 Fig. A total of 125 out of 137 (91.2%) accessions of CTC were assigned to the China tea group, and the remaining 12 samples to the Mosaic group. The CTIN samples showed a high genetic admixture with only 6 out of 41 (14.6%) samples assigned to the China tea group, 5 samples (12.2%) to the Indian Assam tea group, while the majority (30 samples; 73.2%) fell in the Mosaic group. For the ASTC samples, 99 out of 163 (60.7%) accessions were assigned to the Chinese Assam tea group, and the remaining 64 samples (39.3%) to the Mosaic group. While 86.7% (39 out of 45) of the accessions of ASTIN were assigned to the Indian Assam tea group, the remaining six accessions were placed in the Mosaic group. Of the six CAM samples, two were included in the Indian Assam tea group and four accessions were assigned to the Mosaic group. Five samples based on morphology identified as CTIN (T 246, CP 1, T 135, K 1/1, B/5/63) and two identified as CAM (TV 9 and TV 20) were reassigned to the Indian Assam tea group. In total, our molecular analysis assigned 131 samples to the China tea group, 99 to the Chinese Assam tea group, 46 the Indian Assam tea group and 116 fell in the Mosaic group (Table 2).

**Table 2:** Genetic diversity parameters of Chinese and Indian tea after regrouping based on STRUCTURE analysis results.

Group	N	*A*	*Ar*	*Ap*	*Ho*	*He*	*F*is
China tea	131	239	8.786	15	0.638	0.690	0.08
Chinese Assam tea	99	211	7.873	10	0.550	0.637	0.141
Indian Assam tea	46	200	8.546	8	0.690	0.722	0.055
Mosaic group	116	275	10.31	22	0.659	0.793	0.173
Total	392	315	10.198		0.634	0.710	0.014

N: size of the group; *A*: number of alleles; *Ar*: Allelic richness; *Ap*: number of private alleles; *Ho*: observed heterozygosity; *He*: expected heterozygosity; *F*is, inbreeding coefficient

The genetic diversity indices of the regrouped dataset showed that the Mosaic group had the highest expected heterozygosity (*He* = 0.793), followed by Indian Assam tea (0.722). The Mosaic group also had the highest number of private alleles (22) and allelic richness (10.31) (Table 2). The lowest genetic diversity was estimated for Chinese Assam tea (*He* = 0.637). The number of private alleles of the Chinese Assam tea group was reduced from 36 to 10 compared with those of the initial results for ASTC (Table 2).

The pairwise genetic differentiation among all groups was significant (Table 3). China tea and Chinese Assam tea showed the highest differentiation (0.194), followed by China tea and Indian Assam tea (0.156). A significant differentiation (0.141) was also estimated between Chinese Assam tea and Indian Assam tea. Low levels of differentiation, on the other hand, were recorded between the Mosaic group and the other tea groups.

**Table 3:** Pairwise genetic differentiation among tea groups after regrouping based on the STRUCTURE analysis. All genetic differentiations were significant at (P < 0.005).

Groups	China tea	Chinese Assam tea	Indian Assam tea
China tea	-		
Chinese Assam tea	0.194	-	
Indian Assam tea	0.156	0.141	-
Mosaic group	0.065	0.060	0.053

### Genetic relationships among tea cultivars

**Fig 2:**
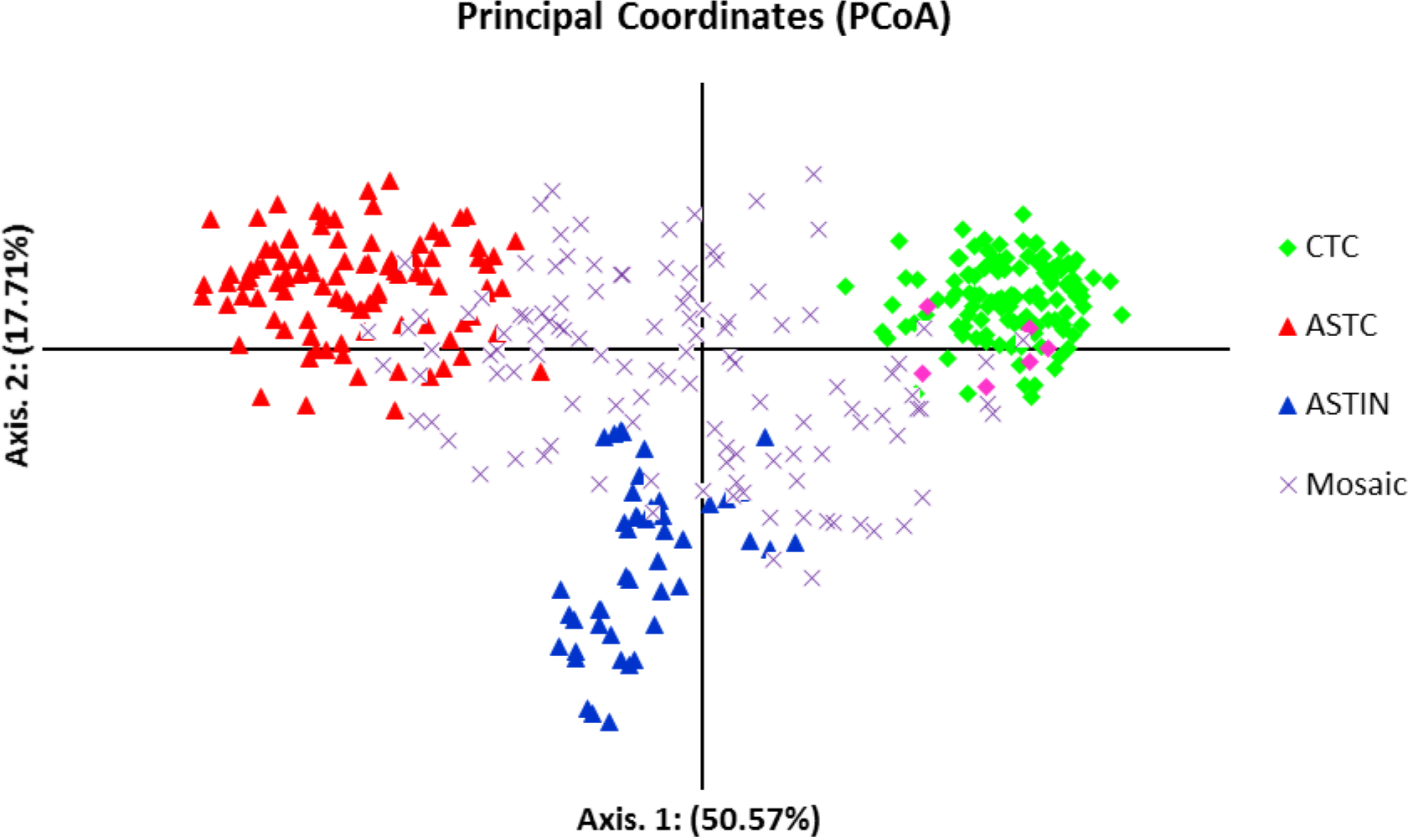
Principal Coordinate Analysis (PCoA) for 392 tea samples collected from China and India after regrouping based on STRUCTURE showing the "pure" and Mosaic tea groups. Pure groups: green diamonds = China tea group, red triangles = Chinese Assam tea group, blue triangles = Indian Assam tea group, × = Mosaic group. Coloring in legend represent group assignation. Pink diamonds denote China tea samples from India clustering with China tea from China.

**Fig 3:**
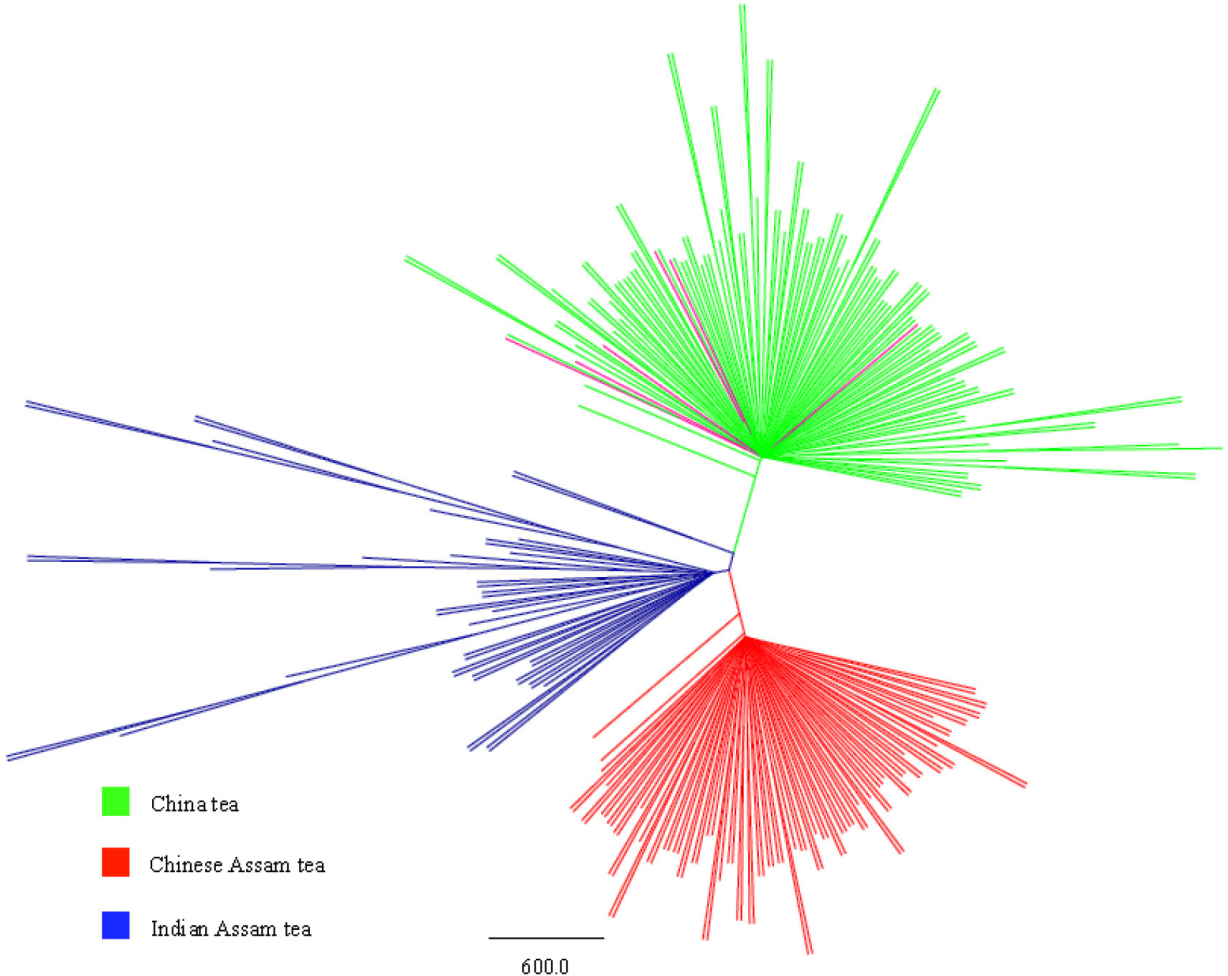
UPGMA tree of three groups of Chinese and Indian tea samples excluding the Mosaic group based on the STRUCTURE analysis. Coloring represents initial type assignation. Pink lines denote China tea samples from India clustering with China tea from China.

Using the regrouped dataset, the first three principal coordinates of the PCoA analysis explained 79.05% of the total variation. Three groups, the China tea, Chinese Assam tea, and Indian Assam tea formed tight and non-overlapping clusters. The accessions of the Mosaic group filled the space between the group clusters with little overlap. Six samples of CTIN fell in the cluster of China tea (Fig 2). The results of the PCoA corresponded well with the grouping based on the STRUCTURE analysis (S3 Fig). The UPGMA tree for the regrouped dataset, excluding samples of the Mosaic group, showed three distinct clusters (Fig 3) consistent with those of the STRUCTURE and PCoA analyses.

## Discussion

### Genetic diversity of tea cultivars from China and India

In general, the genetic diversity is higher in cross-pollinated perennials than in selfing annuals [4]. *Camellia sinensis* is an obligate out-breeder [65], and insect pollinated [66]. In line with this breeding system, high levels of genetic diversity for cultivated tea and its closely wild relatives, have been reported in a number of previous studies [67,39,49]. All the tea types in the present study also showed high levels of genetic diversity (He = 0.71; Range 0.644–0.760) which is consistent with previous studies using SSRs [38,39,49]. However, direct comparison could be misleading since these studies estimated the genetic diversity of tea cultivars based on sample grouping by country or region and not by tea types. For example, the genetic diversity (*Hs*) of 450 tea accessions from China was estimated to be 0.640 [39] which is lower compared to our result. Taniguchi et al [49] on the other hand, reported a much higher genetic diversity (both *He* = 0.850) for tea cultivars from China and India compared to other countries, although they did not separate their samples for tea types. One reason for this discrepancy may be marker bias [49] or the inclusion of a wider gene pool, although both are difficult to quantify given the lack of sampling details associated with their study.

We found that the assignment to tea type based on morphological characters was not always consistent with the genetic groupings based on the STRUCTURE analysis (Fig 1). Morphological features for characterizing tea plants are often influenced by environmental factors resulting in continuous variation and a high degree of plasticity, making accurate identifications sometimes problematic [68,69]. Therefore, molecular techniques have been widely used to overcome the problem of misidentification [69]. In this study, seven tea samples were misidentified but then assigned to the correct group based on molecular data. On the other hand, 116 samples assigned to the Mosaic group showed an admixture of genotypes of different tea types. This may indicate an artificial or spontaneous hybrid origin for these tea cultivars [46] that may be contributing to the misidentifications based on morphology alone. As a consequence of the re-assignments based on the STRUCTURE analysis, the genetic diversity of the re-grouped tea types was lower than that of the tea types initially based on morphological characters (Tables 1 & 2). However, even after regrouping the samples, all groups harbored high levels of genetic diversity. The higher genetic diversity of Indian Assam tea compared to Chinese Assam tea and China tea is likely due to extensive artificial hybridization during its relatively shorter breeding history [46].

### Genetic groupings and the origin of Cambod tea

Genetic clustering by STRUCTURE is a powerful tool to define genetically distinct groups [16,52,59,60], and also used to track recent genetic admixtures [70]. In the present study, three distinct genetic groups were found for all tea accessions from China and India, in addition to a Mosaic group (Figs 2 and 3, S3 Fig). The high genetic differentiation among the three tea groups supports their distinctness. The three tea groups, namely China tea, Chinese Assam tea and Indian Assam tea, corresponded well with their geographic origins (S1 Fig). For CTIN, six samples showing pure genetic material grouped together with China tea, which likely reflect their origin from China. The Cambod type tea has been described based on morphology as a subspecies of *C*. *assamica* [22]. It has morphological features intermediate between the China and Assam types. In this study, the accessions of the Cambod type did not form a distinct group in the STRUCTURE analyses, but showed a mixed genetic composition of Chinese Assam tea and Indian Assam tea with relatively high proportions of the latter (Fig 1). Thus, the Cambod type tea appears to have originated through hybridization between these tea types, and we further demonstrate here that it should not be recognized as a natural taxon [52].

### Indications for three independent domestication events and new insights into the origin of Assam tea

The controversy over the area of origin and center of domestication of the tea plant has existed for a long time. The Sichuan province of China has ever been proposed for its origin [28], or three provinces in Southwest China, Sichuan, Yunnan, and Guizhou [71], or three separate regions, eastern and southeastern China for the China type tea, and Yunnan and Assam in India for the Assam type tea [29]. However, to date there has no strong evidence been presented for any of these hypotheses. In the present study, we defined three distinct genetic groups (Figs 1–3), and each has a distinct different geographical distribution range (S1 Fig). The high genetic divergence between the China type tea (*Camellia sinensis* var. *sinensis*) and the Assam type tea (*C*. *sinensis* var. *assamica*) suggests that they may represent distinct species, which is supported by a recent molecular phylogenic analysis based on complete chloroplast genome sequences [72].

The tea plant was initially used as a medicine and then as a beverage tea in China over 4,000 years [26]. Though it is clear that China is the country of origin for China type tea, no consensus had been reached regarding the specific area of its origin of domestication in China. Eastern China and the Yangtze River region put forward as candidate [29,71]. The “pure” genetic background and polytomous short branches of our UPGMA tree suggest that the domestication of China type tea possibly took place once, and subsequent tea cultivars were developed from that gene pool. Although no truly wild populations of the tea plant of China type tea has ever been found, near-wild tea plants and ancient tea trees have been discovered in mountains of Southern China (e.g. Fujian, Jiangxi, Hunan, and Guizhou), which is a major region for tea production. Thus, we deduce that this region is most likely the area of origin and the origin of domestication of China type tea. Subsequently, China type tea was widely distributed and planted in provinces along the Yangtze River region (S1 Fig). Collectively, our results indicate that China tea in China and India are genetically similar and hence, cultivated China type tea might have been directly introduced from China to India, where it has consecutively undergone extensive hybridization with Indian Assam tea (Fig 1) [73,74].

Furthermore, we found for the first time that Chinese Assam tea is a distinct genetic lineage. Southwest Yunnan and adjacent regions of Indo-China (Myanmar, Assam in India, Northern Thailand, Laos, Vietnam and Cambodia) have also been suggested as an area of origin of the tea plant [33,71,75] and the domestication of Chinese Assam tea in Southwest Yunnan by local people as early as around 2,000 years ago seems likely [76]. The presence of ancient tea plants (some trees estimated over 1000 years old) in this genetic group suggests that Southwest Yunnan probably represents an area of origin and domestication of the Chinese Assam type tea, although to date no wild tea plants of Assam tea in China have been discovered.

The interesting finding in our study was that Assam tea from China is not closely related to that of India. All analyses performed namely; STRUCTURE, PCoA and UPGMA tree (Figs 1–3) clearly show that the Chinese and Indian Assam teas are genetically distinct and showed a significant genetic differentiation (0.141) (Table 3). Compared to China, the recorded cultivation history of Assam type tea is relatively short in India [77]. It has been earlier assumed that Indian Assam tea was introduced from Yunnan, China through Myanmar to India [76], even though it was discovered in Assam, India in 1823 and subsequently cultivated here [31]. Given the short breeding history of this tea in Assam [77], it seems unlikely that plants introduced from China to India would form distinct lineages in both countries. Furthermore, one of the near-wild tea samples from Margherita, India and the cultivated Assam tea from India grouped together genetically, which further suggests that Indian Assam tea originated and was domesticated locally. In addition, historical evidence suggests that the “Singpho” tribe of Margherita, India used indigenous tea from wild plants before tea plants were introduced to Assam [78]. The “Assam race” was known among the hill tribes in Arunachal Pradesh, India, who used the tender leaves to prepare a traditional drink. This was well before the initiation of tea cultivation by the British [77]. Together, the evidence supports a likely independent domestication of Assam type tea in Assam, India, and should be regarded as a distinct genetic lineage from ‘Assam’ tea in China.

## Conclusions

We explored the origin and domestication history of tea plants using molecular markers. Three distinct genetic entities, China tea, Chinese Assam tea and Indian Assam tea, were defined based on genetic clustering in this study of tea plants collected from China and India. Our results are not consistent with the traditional classification of *Camellia sinensis* but indicate that Chinese Assam tea is a distinct genetic linage compared to Assam tea from Assam, India. We further conclude that China type tea, Chinese Assam type tea and Indian Assam type tea were likely domesticated independently in Southern China, Southwest Yunnan Province of China, and the Assam of India, respectively. The newly identified and genetically distinct Chinese Assam tea will be a valuable germplasm resource for future tea breeding, and the ancient tea plants of Chinese Assam tea should be a high conservation priority.

## Supporting Information

S1 Fig Geographic distribution of tea cultivars analyzed in the current study according to the collection provinces from China and India. (PDF)

S2 Fig Results of detecting best *K* value based on Δ*K* (A) and Log Likelihood (B) methods.
(PDF)

S3 Fig Results of the STRUCTURE analysis after regrouping. (PDF)

S1 Table Collection information of the 392 tea samples used in the current study. (XLS)

S2 Table Description of Microsatellite loci. (PDF)
