# Supplementary material for: Indications for Three Independent Domestication Events for the Tea Plant (Camellia sinensis (L.) O. Kuntze) and New Insights into the Origin of Tea Germplasm in China and India Revealed by Nuclear Microsatellites
Source: PLoS One. 2016 May 24;11(5):e0155369. doi: 10.1371/journal.pone.0155369 (PMC4878758; doi:10.1371/journal.pone.0155369)

**S1 Fig: Geographic distribution of tea cultivars analyzed in the current study according to the collection provinces from China and India**

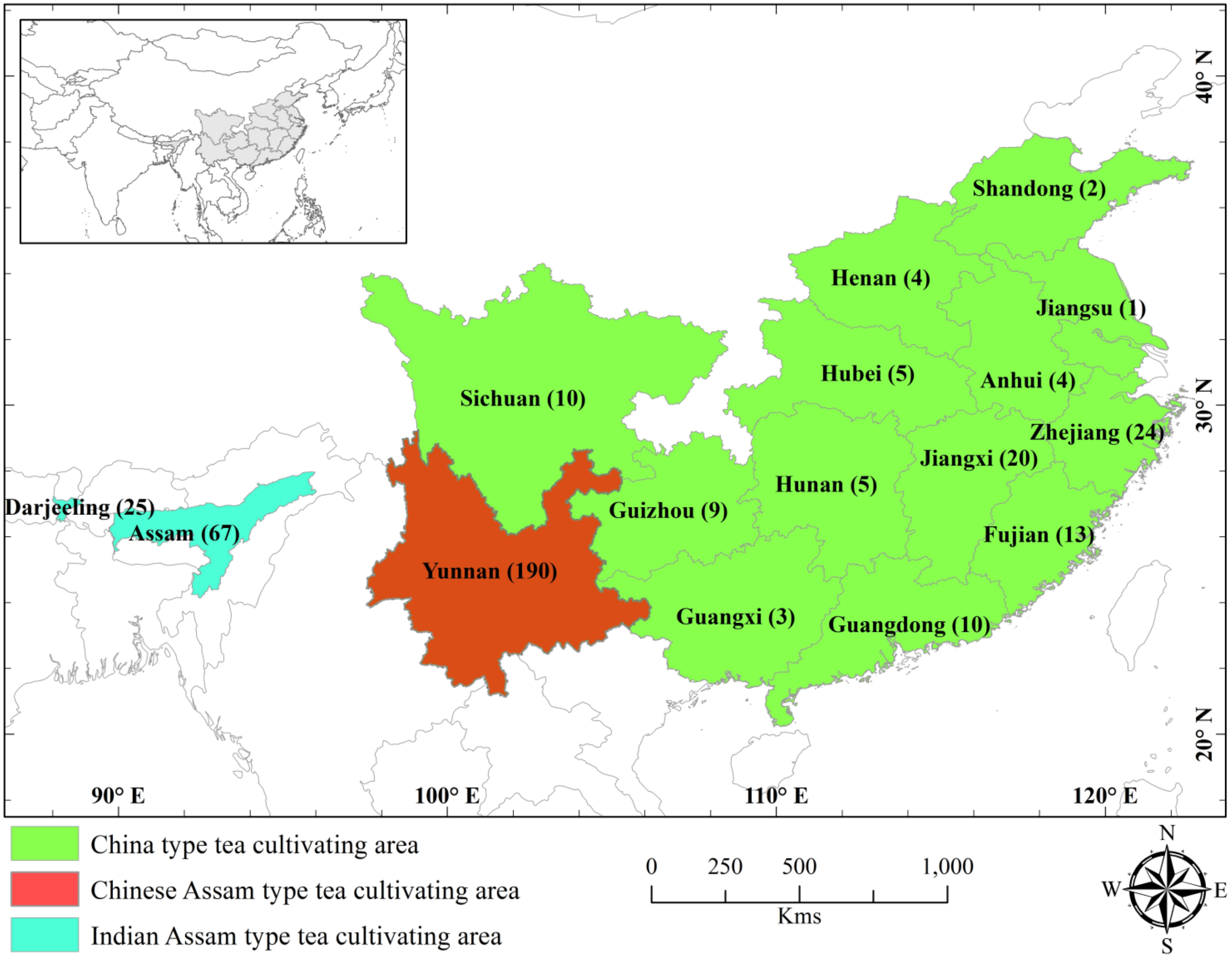

Supplement: S1 Fig — (PDF) [file pone.0155369.s001.pdf]
