# Supplementary material for: Indications for Three Independent Domestication Events for the Tea Plant (Camellia sinensis (L.) O. Kuntze) and New Insights into the Origin of Tea Germplasm in China and India Revealed by Nuclear Microsatellites
Source: PLoS One. 2016 May 24;11(5):e0155369. doi: 10.1371/journal.pone.0155369 (PMC4878758; doi:10.1371/journal.pone.0155369)

S2A Fig: Best *K* value for structure analysis with ΔK method

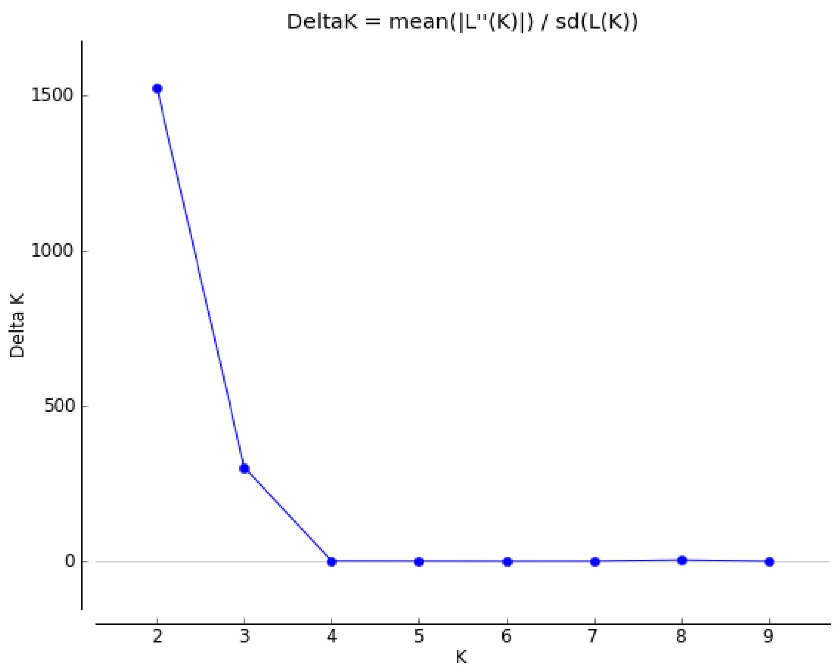

S2B Fig: Best *K* value for structure analysis with Log Likelihood method

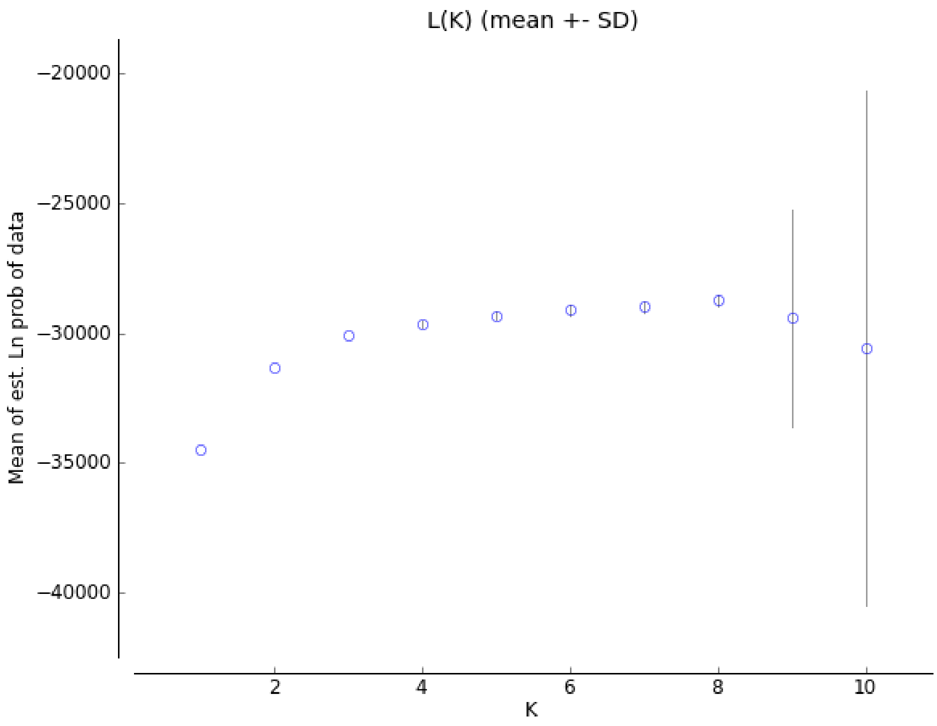

Supplement: S2 Fig — Results of detecting best K value based on ΔK (A) and Log Likelihood (B) methods. (PDF) [file pone.0155369.s002.pdf]
