# Supplementary material for: Indications for Three Independent Domestication Events for the Tea Plant (Camellia sinensis (L.) O. Kuntze) and New Insights into the Origin of Tea Germplasm in China and India Revealed by Nuclear Microsatellites
Source: PLoS One. 2016 May 24;11(5):e0155369. doi: 10.1371/journal.pone.0155369 (PMC4878758; doi:10.1371/journal.pone.0155369)
Supplement: S3 Fig — (PDF) [file pone.0155369.s003.pdf]

S3 Fig: Results of the STRUCTURE analysis after regrouping

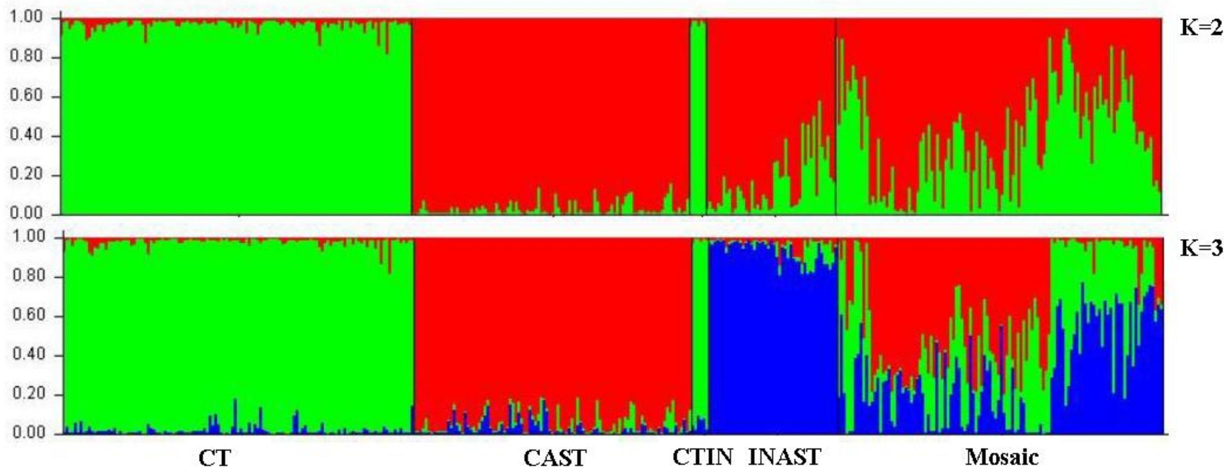

Results of structure analysis for *K*=2 and *K*=3 for the regrouped dataset of 392 tea samples collected from China and India. Abbreviations: CT: China tea; CAST: Chinese Assam tea; CTIN: China tea in India; INAST: Indian Assam tea; Mosaic: Mosaic group
