# Supplementary material for: Indications for Three Independent Domestication Events for the Tea Plant (Camellia sinensis (L.) O. Kuntze) and New Insights into the Origin of Tea Germplasm in China and India Revealed by Nuclear Microsatellites
Source: PLoS One. 2016 May 24;11(5):e0155369. doi: 10.1371/journal.pone.0155369 (PMC4878758; doi:10.1371/journal.pone.0155369)
Supplement: S2 Table — (PDF) [file pone.0155369.s005.pdf]

S2 Table: Description of the Microsatellite loci

No.	Primer Name	Sequence (5'-3')	Size	Repeat	Ta (°C)	Reference
1	Po9	F-CAGGGTTGCAAGAAGTACCG	122	(TTC)n	65 C	[1]
		R-ATCAACCGTATGGGCAAAAG				
2	Ca8	F-TTCAATTACCCGCCAATCTC	193-255	(CT)10	58 C	[2]
		R-CCAATCTGGGAATTGAAGAAG				
3	A9	F-TTTTATGTAGGGTTTCCGTTCC	124	(TC)13	60 C	[3]
		R-GTCGTACTTGCGCTTGTTGG				
4	A37	F-TCTGCCCTTCCCTAAATC	170-182	(AAG)9	53 C	[4]
		R-ATGTTTGGTCTCGGTTGTT				
5	A47	F-TCCCTACAAACCCTAACCG	185	(GCC)6	61 C	[3]
		R-GAGCAGCATCAGAGTCACGT				
6	A87	F-AGGGGACGGATCTCATATCGT	202	(AG)11	58 C	[3]
		R-GACTTCCTCACCGGAGTGCTT				
7	Q6	F-CATTACCTCCAATCTCCG	229–263	(AG)23	56 C	[4]
		R-CACGCTCATCTCCTCTTT				
8	TUGMS2-135	F-ATGCTAGCCATGGCAATACC	245-300	(GAA)8	56 C	[5]
		R-CACACTGCACATGATGGTGA				
9	TUGMS2-143	F-TCTCGTTGAGGGGAGAAGTC	235–270	(TTC)14	54.5 C	[5]
		R-GAGGAGCGAGAGATGGGTTT				
10	TUGMS2-157	F-CCCATGGTCTATTTCGCTGT	165–180	(CCA)16	53 C	[5]
		R-CCAGAGATGGACCTGACACA				
11	TM51	F-AATCATGCCCAAGGACATTC	170-180	(GGT)6	60 C	[6]
		R-CAACCACTACCCATTTCACT				
12	TM58	F-CATTATCCCTTTCCTTGTCCA	250–270	(TCA)6	61 C	[6]
		R-GGAGGGAGTAGGAGGTCT				
13	TM 134	F-TTCCGTGACTGATTTATGTG	230–260	(CAT)8	56 C	[6]
		R-TTGAGACTCGGGGTTTT				
14	TM148	F-GCGTCTGCTGCCGAAAT	135-155	(ACC)8	63 C	[6]
		R-ACAAACATCCTCCTCACCC				

15	TM179	F-GTCCCAGAAATCATAACG	148–170	(TGA)8	58 C	[6]
		R-CGACAAGGGATTAGCAG				
16	TM197	F-GAGGAGCATTAGCATCTT	120–150	(AGG)7	59 C	[6]
		R-GGACCAGTACGAGTAGC				
17	TM203	F-AGAGCTTCTCAACAACCC	165-200	(GAT)9	57 C	[6]
		R-ATGGAGCATACTACTCACTT				
18	S34	F-CTGAGGCTCATCAACGCATA	136	(AAG)15	61 C	[7]
		R-GCATCTGTACAGCTCCTCCC				
19	S53	F-TGTTGCTGAGACAAATTCGC	217	(GGC)5	58 C	[7]
		R-AATGGCGTCTGGGACATTAG				
20	S71	F-CGACTCTTGACTCCTCTCTGC	207	(AAC)7	56 C	[7]
		R-AGTCGGACTGGGAATAGGCT				
21	S76	F-GAGAAACAACAATAAAATGGAGGC	233	(ATA)9	62 C	[7]
		R-AACCAGACGTTTGGAGCAAC				
22	S80	F-TATTCCCACCACTCCTGCCT	210	(ATG)9	58 C	[7]
		R-CGACGATGATTACGAGGACA				
23	S87	F-GTATTGGGAGCGCAAGATCA	133	(CAACAG)6	60 C	[7]
		R-GAGTCTTGACGGAGTCGAGG				

References:

1. Jin J.Q, Cui HR, Chen WY, Lu M.Z, Yao YL, Xin Y, Gong XC. Data mining for SSRs in ESTs and development of EST-SSR marker in tea plant (*Camellia sinensis*). J Tea Sci 2006; 26(1):17–23

2. Hung CY, Wang KH, Huang CC, Gong X, Ge XJ, Chiang TY. Isolation and characterization of 11 microsatellite loci from *Camellia sinensis* in Taiwan using PCR-based isolation of microsatellite arrays (PIMA). Conserv Genet 2008; 9:779–781

3. Wang LY, Jiang YH, Duan YS, Cheng H, Zhou J, Zeng JM. Characterization of EST-derived Microsatellites and Development of SSR-markers in Tea (*Camellia sinensis*) primers. J Plant Genet Resour 2009; 10(4):511–516
4. Yang JB, Yang J, Li HT, Zhao Y Yang SX. Isolation and characterization of 15 microsatellite markers from the wild tea plant (*Camellia taliensis*) using FIASCO method. Conserv Genet 2009; 10:1621–1623
5. Sharma H, Kumar R, Sharma V, Kumar V, Bhardwaj P, Ahuja PS, Sharma RK. Identification and cross-species transferability of 112 novel UniGene-derived microsatellite markers in tea (*Camellia sinensis*). Am J Bot 2011; 98(6):133–138
6. Yao MZ, Ma CL, Qiao TT, Jin JQ, Chen L. Diversity distribution and population structure of tea germplasms in China revealed by EST-SSR markers. Tree Genet Genomes 2012; 8: 205–220
7. Wu H, Chen D, Li J, Yu B, Qiao X, Huang H, He Y. De novo characterization of leaf transcriptome using 454 sequencing and development of EST-SSR markers in tea (*Camellia sinensis*). Plant Mol Biol Rep 2012; 31(3):524–538
